# Supplementary material for: Partitioned polygenic scores show mechanistic heterogeneity in type 2 diabetes and hypertension comorbidity
Source: Nat Commun. 2026 Feb 9;17:1446. doi: 10.1038/s41467-025-67449-2 (PMC12886974; doi:10.1038/s41467-025-67449-2)
Supplement: Supplementary file 1 — Supplementary Information [file 41467_2025_67449_MOESM1_ESM.pdf]

Partitioned polygenic scores show mechanistic heterogeneity in type 2 diabetes and hypertension comorbidity

Supplementary Information

1. Supplementary Figures

Supplementary Figure 1: Hierarchical clustering of the 1,304 SNVs using *pheatmap*. 3
Supplementary Figure 2: Comparison of the unadjusted vs adjusted Z-score strategies for hierarchical clustering. a, Hierarchical clustering of the 1,304 variants with unadjusted Z-scores (original figure 2). **b,** Hierarchical clustering of the 1,304 variants with adjusted Z-scores**. c,** Contingency heatmap of the assignment of the 1,304 variants in the non-adjusted vs adjusted clustering (Chi-squared test of independence $p<2x10^{-16}$). 5
Supplementary Figure 3: *MRClust* results using T2D as exposure and PP as outcome using the curated 563 T2D SNVs as instrument variables (***Methods – Clusters of pathogenetic processes***). 6
Supplementary Figure 4: Bayesian nonnegative matrix factorization (bNMF) clustering using the 1,304 T2D-BP SNVs, ran with default parameters and 10 iterations (***Methods – Clusters of pathogenetic processes***). Most iterations (5/10) resulted in 8 groups. **a,** Variant association to clusters. **b,** Feature (GWAS summary statistics) association to clusters. **c,** Comparison between the hierarchical clusters (columns) and the bNMF clusters (rows). Each value intensity represents the associated -log(P-value) of the linear association while the colour is the direction of the linear association beta (***Methods – Clusters of pathogenetic processes***). 7
Supplementary Figure 5: a, Bar plot comparing SNVs attributed to the T2D-BP hierarchical clusters (x-axis) and the latest T2D hierarchical clustering from Suzuki et al. (2024). **b,** Bar plot comparing SNVs attributed to the T2D-BP hierarchical clusters (x-axis) and the latest T2D 'soft' clustering from Smith et al. (2024). **c,** Sankey plot comparing SNV attributed to the T2D-BP hierarchical clusters (left) and the latest T2D hierarchical clustering from Suzuki et al. (right). **d,** Sankey plot comparing SNV attributed to the T2D-BP hierarchical clusters (left) and the latest T2D 'soft' clustering from Smith et al. (right). The comparison is done using each study genetic variants and looking for LD proxy (LD r^2 > 0.6) in our T2D-BP genetic variants. For 'soft' clusters, the cluster assignment is based on weight > 0.75 (***Methods – Clusters of pathogenetic processes***). 9
Supplementary Figure 6: a, Bar plot comparing SNVs attributed to T2D-BP hierarchical clusters (x-axis) and the latest BP 'soft' clustering from Vaura et al. (2022). **b,** Sankey plot

comparing SNV attributed to the T2D-BP hierarchical clusters (left) and the latest BP ‘soft’ clustering from Vaura et al. (right). The comparison is done using each study genetic variants and looking for LD proxy (LD r^2 > 0.6) in our T2D-BP genetic variants. For ‘soft’ clusters, the cluster assignment is based on weight > 0.75 (***Methods – Clusters of pathogenetic processes***). 10

Supplementary Figure 7: GWAS weight comparison between the hierarchical clusters (columns) and the cluster of the T2DGGI paper (rows). Each value intensity represents the associated Pearson correlation coefficient (***Methods – Clusters of pathogenetic processes***). 11

Supplementary Figure 8: Heat map of colocalised loci across the five clusters and 50 human adult tissues. Each column corresponds to a cluster, while each row represents a tissue. The numerical value in each tile indicates the number of colocalised loci for a specific cluster and tissue. Colour intensity corresponds to the percentage of colocalisation per cluster. 12

Supplementary Figure 9: Pathway analysis of the 202 colocalised genes within the *Inverse T2D-BP risk* cluster using *metascape*. The following genes were associated with the pathway mentioned above. 13

Supplementary Figure 10: Diagram of criteria used in the UKB to identify T2D and hypertension cases, and adjust BP levels. 14

Supplementary Figure 11: Cumulative hazard plot of T2D-BP comorbidity stratified by being in the top 33 percent of the unweighted partitioned PGS after clustering alongside traditional P+T weighted PGS for T2D (magenta) and SBP (sage) with 95% CI. 15

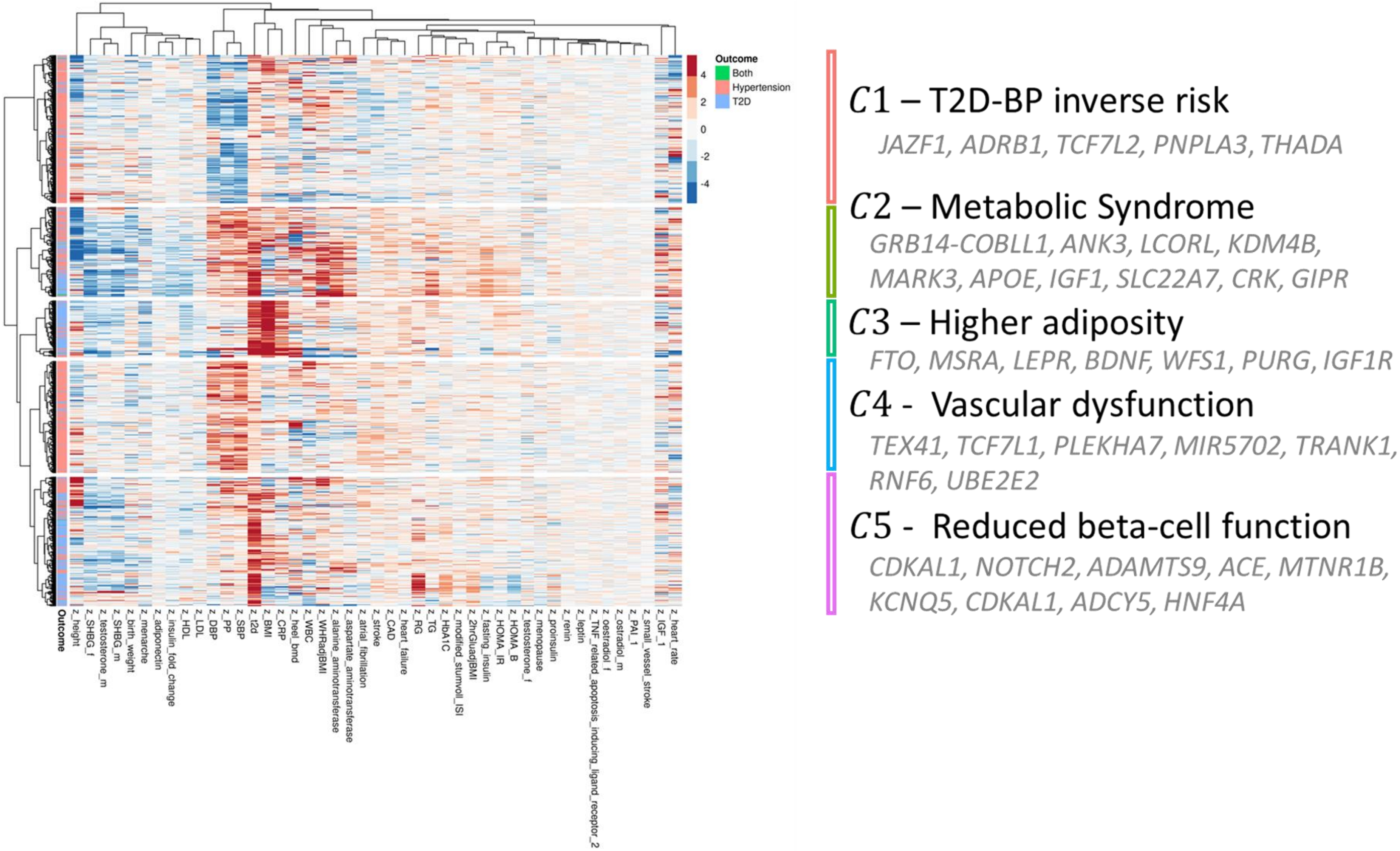

Supplementary Figure 1: Hierarchical clustering of 1,304 SNVs using *pheatmap*.

Legend:

Each row is a SNV, each column is a GWAS of one named phenotype; effect estimates are reported in z-scores = beta/se.

The first column represents the origin of the SNV (from T2D GWAS in blue, BP GWAS in red or both in green).

The black text to the right of the clustering is the attributed cluster names. The grey text represents associated loci within the cluster.

T2D = Type 2 Diabetes; DBP = Diastolic Blood Pressure; UKB = UK Biobank; PP = Pulse Pressure; SBP = Systolic Blood Pressure; HbA1C = Glycated hemoglobin; RG = Random glucose; WHR = waist-hip ratio; BMI = body mass index; HDL = high-density lipoprotein; PAI = Plasminogen activator inhibitor; ISI = Insulin Sensitivity Index; IGF = Insulin-like growth factor; LDL = low-density cholesterol; adjBMI = adjusted for BMI; HOMA = homeostatic model assessment; IR = insulin resistance; B = beta-cell function; WBC = white blood cell count; CRP = C-reactive protein; CAD = coronary artery disease; TG = triglycerides; SHBG = sex-hormone-binding globulin; TRAIL-R2 = TNF related apoptosis inducing ligand_receptor 2; ALT = alanine aminotransferase; AST = aspartane aminotransferase; BMD = bone mass density; TSH = thyroid stimulating hormone; FT4 = free thyroxine

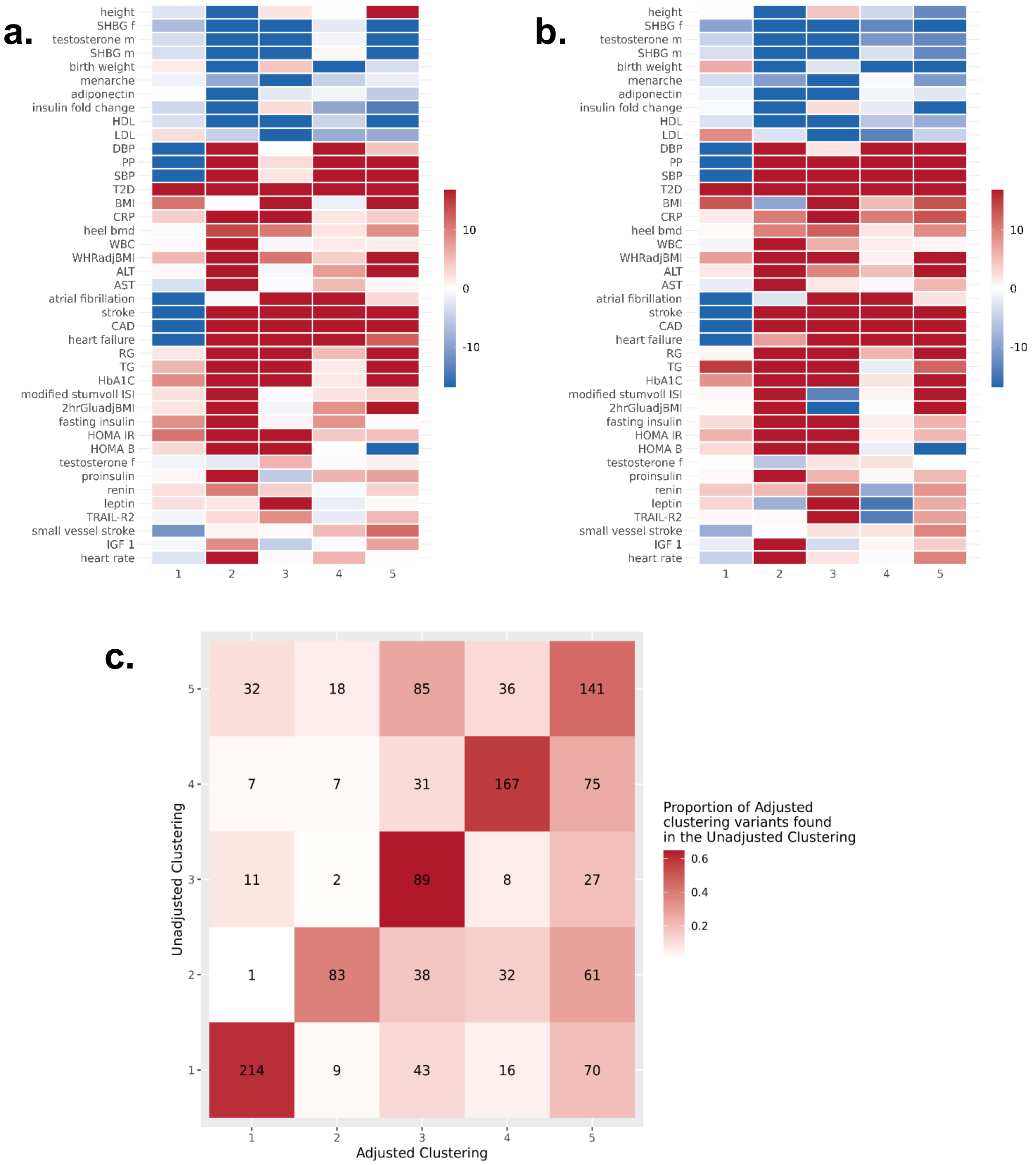

Supplementary Figure 2: Comparison of unadjusted vs adjusted Z-score strategies for hierarchical clustering. a, Hierarchical clustering of 1,304 variants with unadjusted Z-scores (original figure 2). Colour intensity represents the significance, expressed as -log(P-value) from two-sided t-tests on the regression coefficients linking SNV cluster assignment with GWAS effect sizes. **b,** Hierarchical clustering of 1,304 variants with adjusted Z-scores. **c,** Contingency heatmap of the assignment of 1,304 variants in non-adjusted vs adjusted clustering (two-sided Chi-squared test of independence $p<2x10^{-16}$).

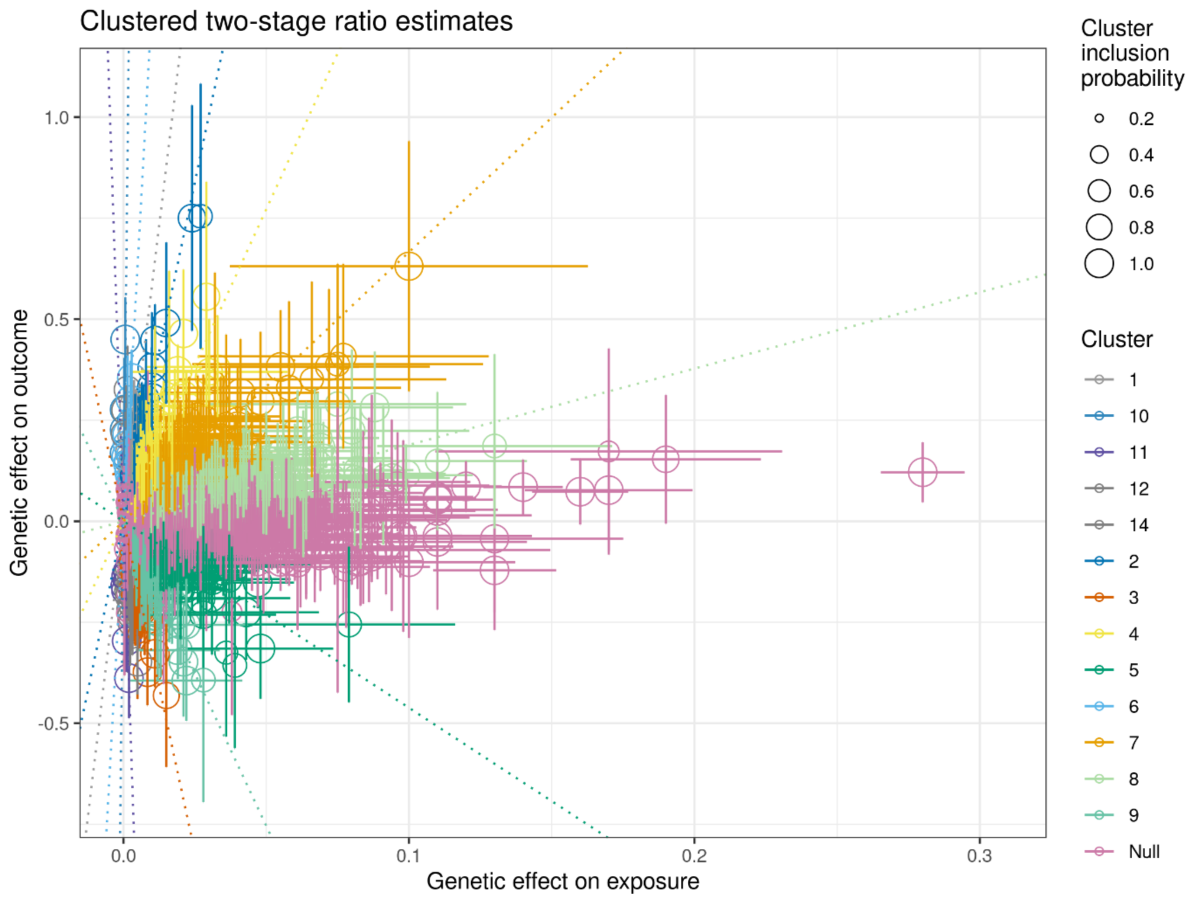

Supplementary Figure 3: *MRClust* causality analysis. Mendelian Randomization results using *MRClust* to partitioned the effect of both exposure and outcome. This figure shows T2D as exposure and PP as outcome using the curated 563 T2D SNVs as instrument variables (***Methods – Clusters of pathogenetic processes***).

Legend:

T2D = Type 2 Diabetes; PP = Pulse Pressure; SNV = single nucleotide variants; MR = Mendelian randomization

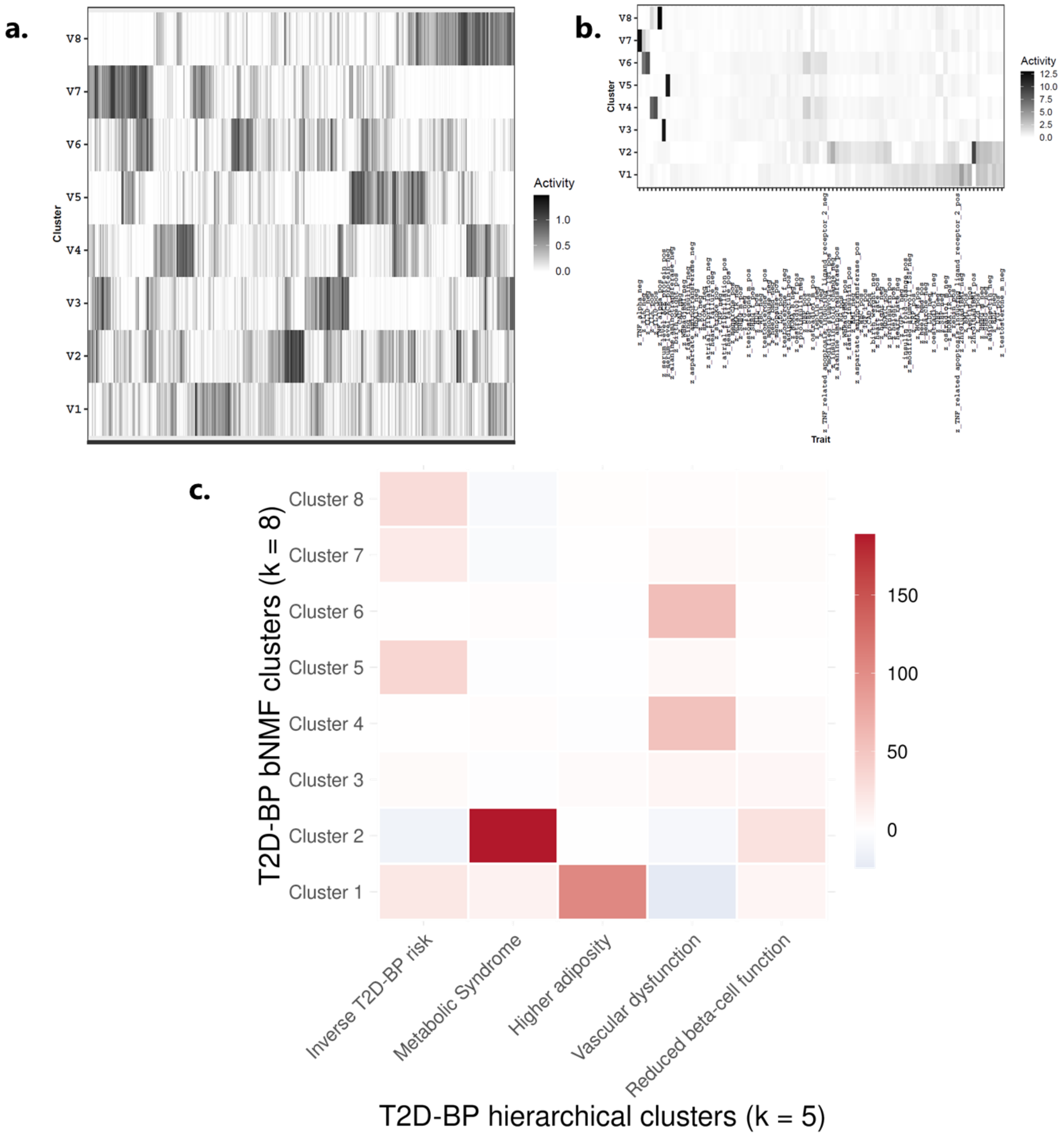

Supplementary Figure 4: Bayesian nonnegative matrix factorization (bNMF) clustering using 1,304 T2D-BP SNVs, ran with default parameters and 10 iterations (*Methods – Clusters of pathogenetic processes*). Most iterations (5/10) resulted in 8 groups. **a,** Variant association to clusters. **b,** Feature (GWAS summary statistics) association to clusters. **c,** Comparison between the hierarchical clusters (columns) and the bNMF clusters (rows). Each value intensity represents the associated -log(P-value) of the logistic regression (hierarchical cluster ~ bNMF weight) while the colour is the sign of the associated beta coefficient (***Methods – Clusters of pathogenetic processes***).

<u>Legend:</u>
bNMF = Bayesian nonnegative matrix factorization; T2D = Type 2 Diabetes; DBP = Diastolic Blood Pressure; UKB = UK Biobank; PP = Pulse Pressure; SBP = Systolic Blood Pressure; HbA1C = Glycated hemoglobin; RG = Random glucose; WHR = waist-hip ratio; BMI = body

mass index; IL = interleukin; HDL = high-density lipoprotein; PAI = Plasminogen activator inhibitor; ISI = Insulin Sensitivity Index; IGF = Insulin-like growth factor; LDL = low-density cholesterol; adjBMI = adjusted for BMI; HOMA = homeostatic model assessment; IR = insulin resistance; B = beta-cell function; WBC = white blood cell count; CRP = C-reactive protein; CAD = coronary artery disease; TG = triglycerides; SHBG = sex-hormone-binding globulin; ACE = Angiotensin-converting enzyme; TRAIL-R2 = TNF related apoptosis inducing ligand_receptor 2; ALT = alanine aminotransferase; AST = aspartane aminotransferase; BMD = bone mass density; TSH = thyroid stimulating hormone; FT4 = free thyroxine

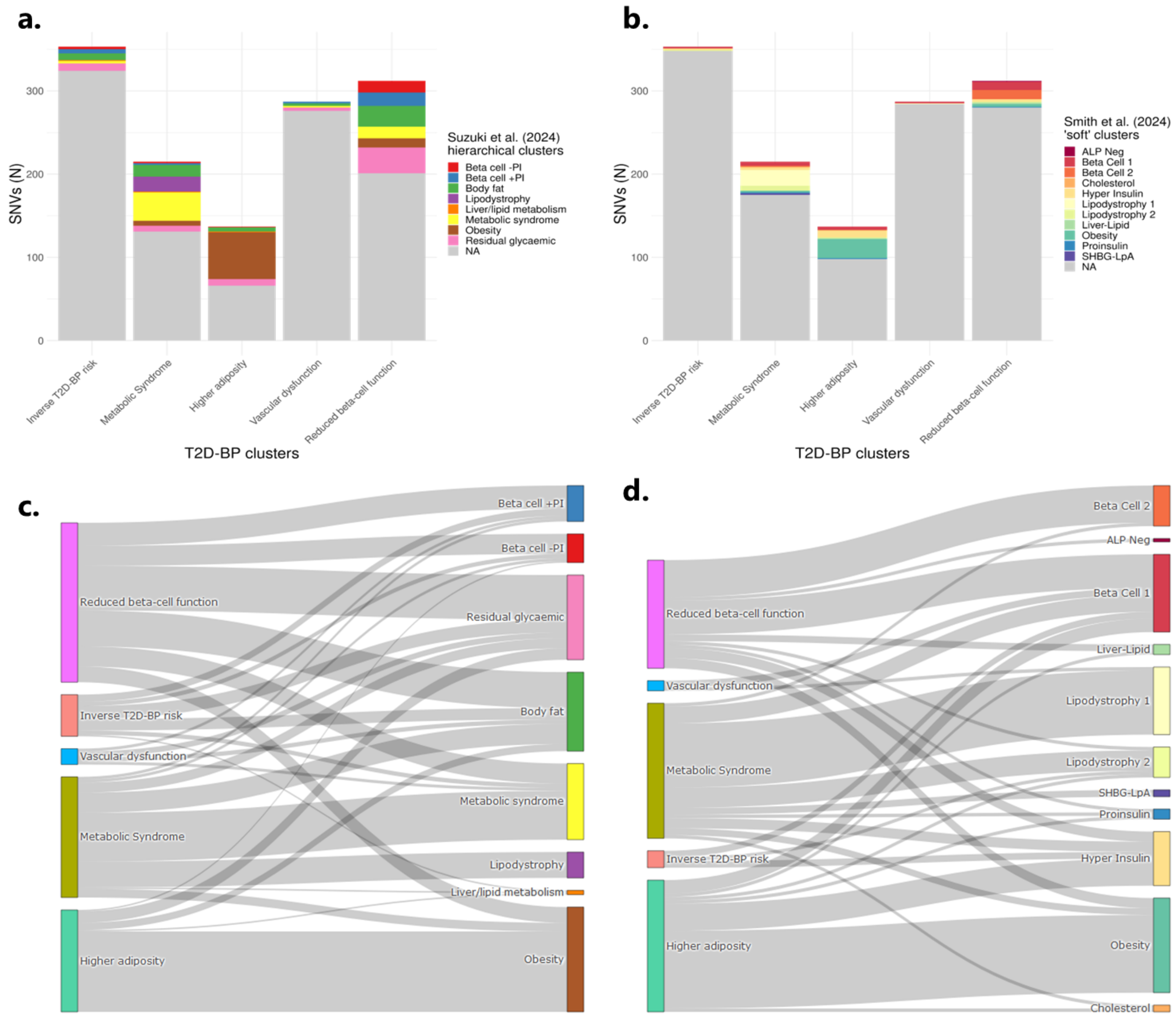

Supplementary Figure 5: Comparison of SNVs assigned to clusters defined in this study with those from the hierarchical clusters from Suzuki *et al.* (2024) and the 'soft' clusters from Smith *et al.* (2024). a, Bar plot comparing SNVs attributed to the T2D-BP hierarchical clusters (x-axis) and the latest T2D hierarchical clustering from Suzuki et *al*. (2024). **b,** Bar plot comparing SNVs attributed to the T2D-BP hierarchical clusters (x-axis) and the latest T2D 'soft' clustering from Smith et *al*. (2024). **c,** Sankey plot comparing SNV attributed to the T2D-BP hierarchical clusters (left) and the latest T2D hierarchical clustering from Suzuki et *al*. (right). **d,** Sankey plot comparing SNV attributed to T2D-BP hierarchical clusters (left) and the latest T2D 'soft' clustering from Smith et *al*. (right). The comparison is done using each study genetic variants and looking for LD proxy (LD $r^2 > 0.6$) in our T2D-BP genetic variants. For 'soft' clusters, the cluster assignment is based on weight > 0.75 (***Methods – Clusters of pathogenetic processes***).

Legend:

T2D = Type 2 Diabetes; BP = Blood Pressure; PI = Proinsulin ; NA = Non attributed; ALP = alkaline phosphatase ; SHBG = sex hormone binding globulin; LpA = lipoprotein A; SNV = single nucleotide variant.

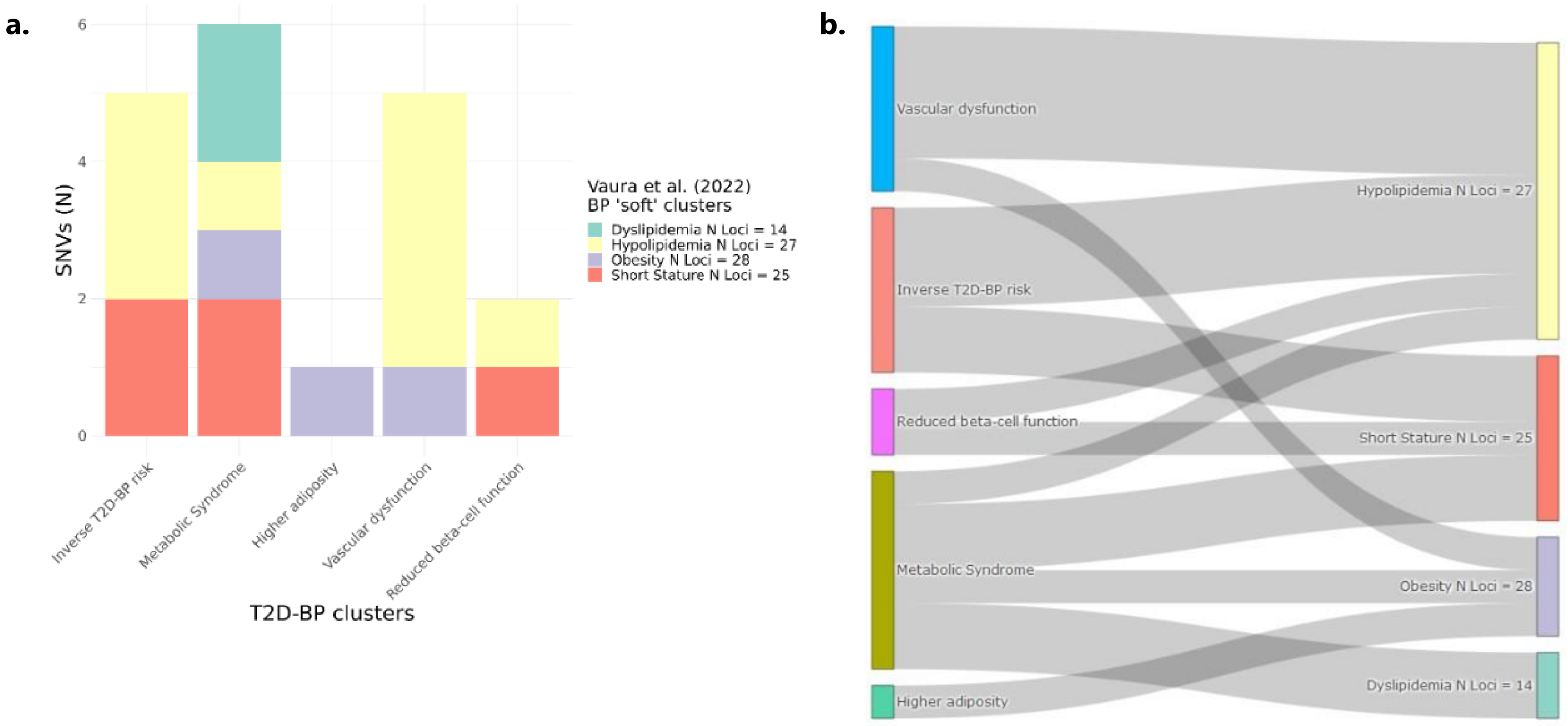

Supplementary Figure 6: Comparison of SNVs assigned to clusters, defined in this study, with those from the 'soft' clusters of Vaura *et al.* (2022). a, Bar plot comparing SNVs attributed to T2D-BP hierarchical clusters (x-axis) and the latest BP 'soft' clustering from Vaura et al. (2022). **b,** Sankey plot comparing SNV attributed to the T2D-BP hierarchical clusters (left) and the latest BP 'soft' clustering from Vaura et al. (right). The comparison is done using each study genetic variants and looking for LD proxy (LD $r^2 > 0.6$) in our T2D-BP genetic variants. For 'soft' clusters, the cluster assignment is based on weight > 0.75 (***Methods – Clusters of pathogenetic processes***).

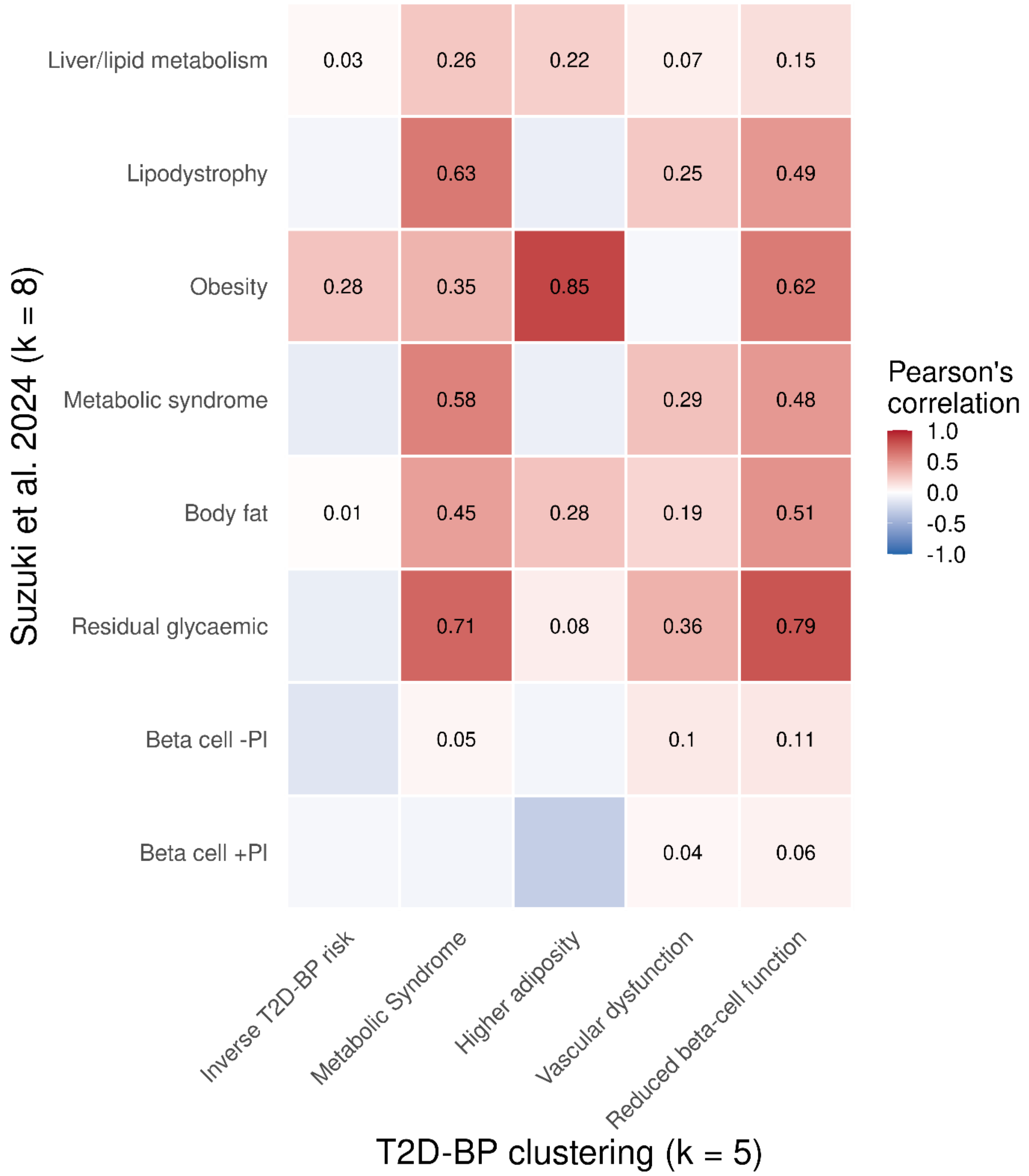

Supplementary Figure 7: GWAS weight comparison between hierarchical clusters from this study (columns) and the cluster from the T2DGGI paper (rows). Each value intensity represents the associated Pearson correlation coefficient (***Methods – Clusters of pathogenetic processes***).

Legend:
T2D = Type 2 Diabetes; BP = Blood Pressure; PI = Proinsulin

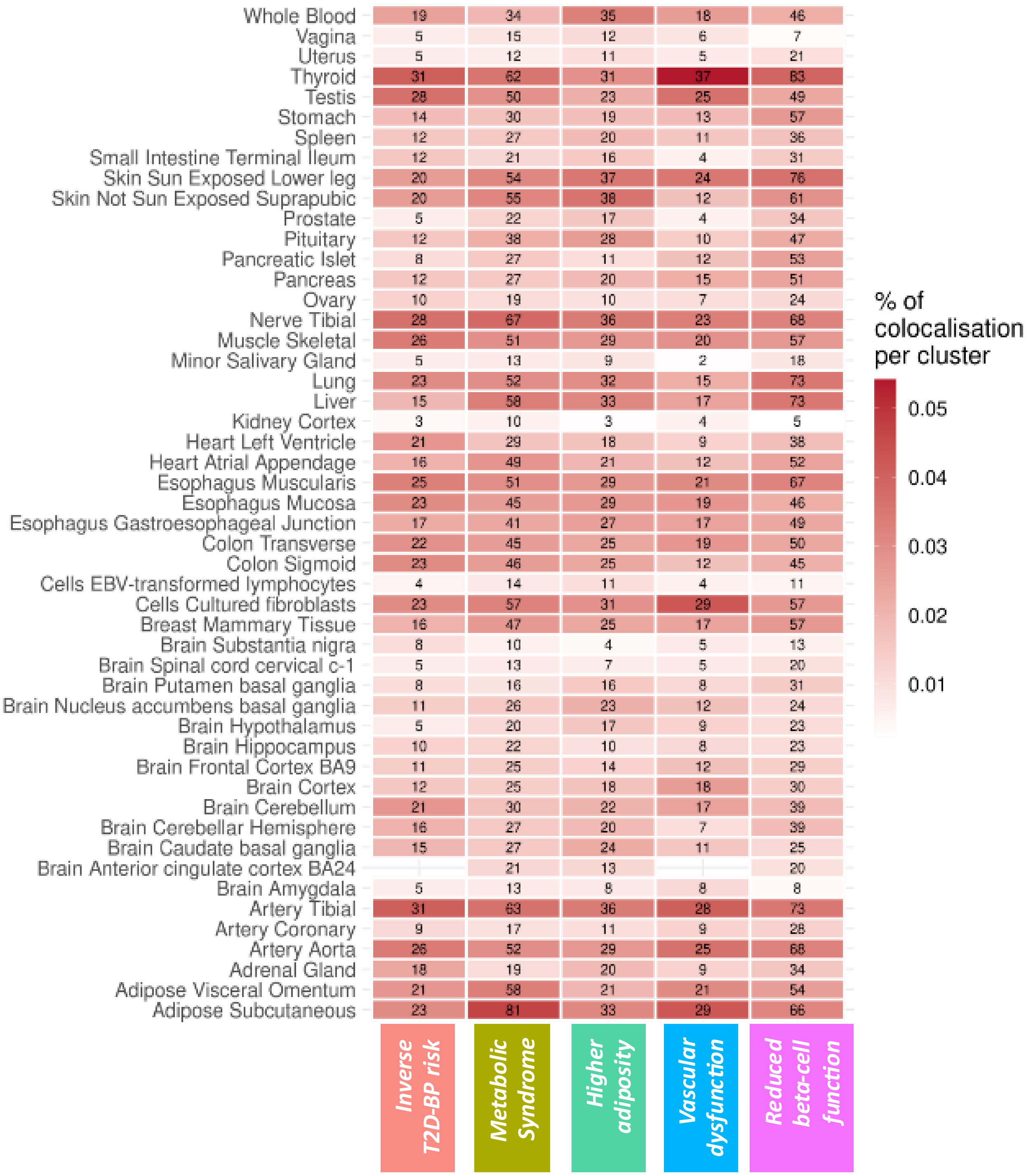

Supplementary Figure 8: Heat map of colocalised loci across five clusters of T2D-BP genetic variant and 50 human adult tissues. Each column corresponds to a cluster, while each row represents a tissue. The numerical value in each tile indicates the number of colocalised loci (using Bayesian colocalization, two-sided) for a specific cluster and tissue. Colour intensity corresponds to the percentage of colocalisation per cluster.

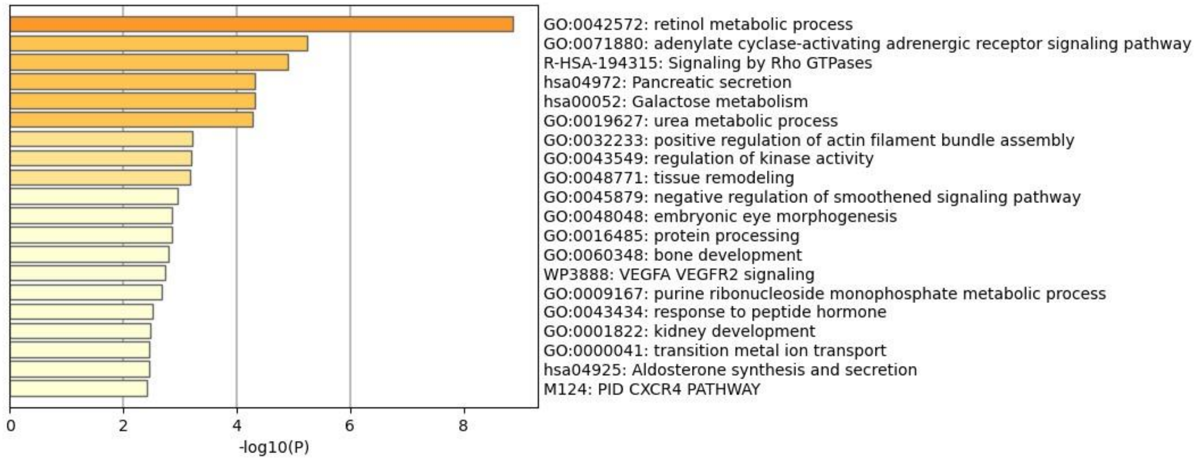

Supplementary Figure 9: Pathway analysis of 202 colocalised genes within the *Inverse T2D-BP risk* cluster using *metascape*. The following genes were associated with the pathway mentioned above.

Category	GO	Description	Colocalised genes associated
GO Biological Processes	GO:0042572	retinol metabolic process	AKR1B1|CYP1A1|CYP2C8|CYP2C18|AKR1B10|RDH14|PLB1|AKR1B15
GO Biological Processes	GO:0071880	adenylate cyclase-activating adrenergic receptor signaling pathway	ADCY9|ADRB1|PLN|AKAP13
Reactome Gene Sets	R-HSA-194315	Signaling by Rho GTPases	RHOC|ARHGAP1|CDC20|CSK|PTK2|FERMT2|AKAP13|SPEN|SWAP70|MCF2L|NUP160|EVL|PLEKHG1|EFHD2|OBSCN|CENPS
KEGG Pathway	hsa04972	Pancreatic secretion	ADCY9|ATP2B1|PRSS3|SLC4A2|CELA2B|CELA2A
KEGG Pathway	hsa00052	Galactose metabolism	AKR1B1|AKR1B10|G6PC3|AKR1B15
GO Biological Processes	GO:0019627	urea metabolic process	CYP2C9|AGMAT|NAGS

T2D	Self-reported having T2D, verbal interview<br>OR<br>Taking medication related to T2D<br>OR<br>Diabetes diagnosed by doctor and age diabetes diagnosed > 35 and having not started insulin within the one-year diagnosis of diabetes<br>OR<br>HbA1c > 6.4%<br>OR<br>Random glucose > 11.1 mmol/l<br>OR<br>T2D is reported without T1D and gestational diabetes in ICD10 disease code	40,619
DBP	Taking the mean value of automated measurements and manual measurements<br>AND<br>Adjusting by 10 mmHg if taking blood pressure lowering medication	N/A
PP	SBP-DBP	N/A
SBP	Taking the mean value of automated measurements and manual measurements<br>AND<br>Adjusting by 15 mmHg if taking blood pressure lowering medication	N/A
Hypertension	SBP ≥ 150 mmHg<br>OR<br>DBP ≥ 90 mmHg<br>OR<br>Taking blood pressure lowering medication	230,737

Supplementary Figure 10: Diagram of criteria used in the UKB to identify T2D and hypertension cases, and adjust BP levels.

Legend:
T2D = Type 2 Diabetes; BP = Blood Pressure; DBP = Diastolic Blood Pressure; UKB = UK Biobank; PP = Pulse Pressure; SBP = Systolic Blood Pressure

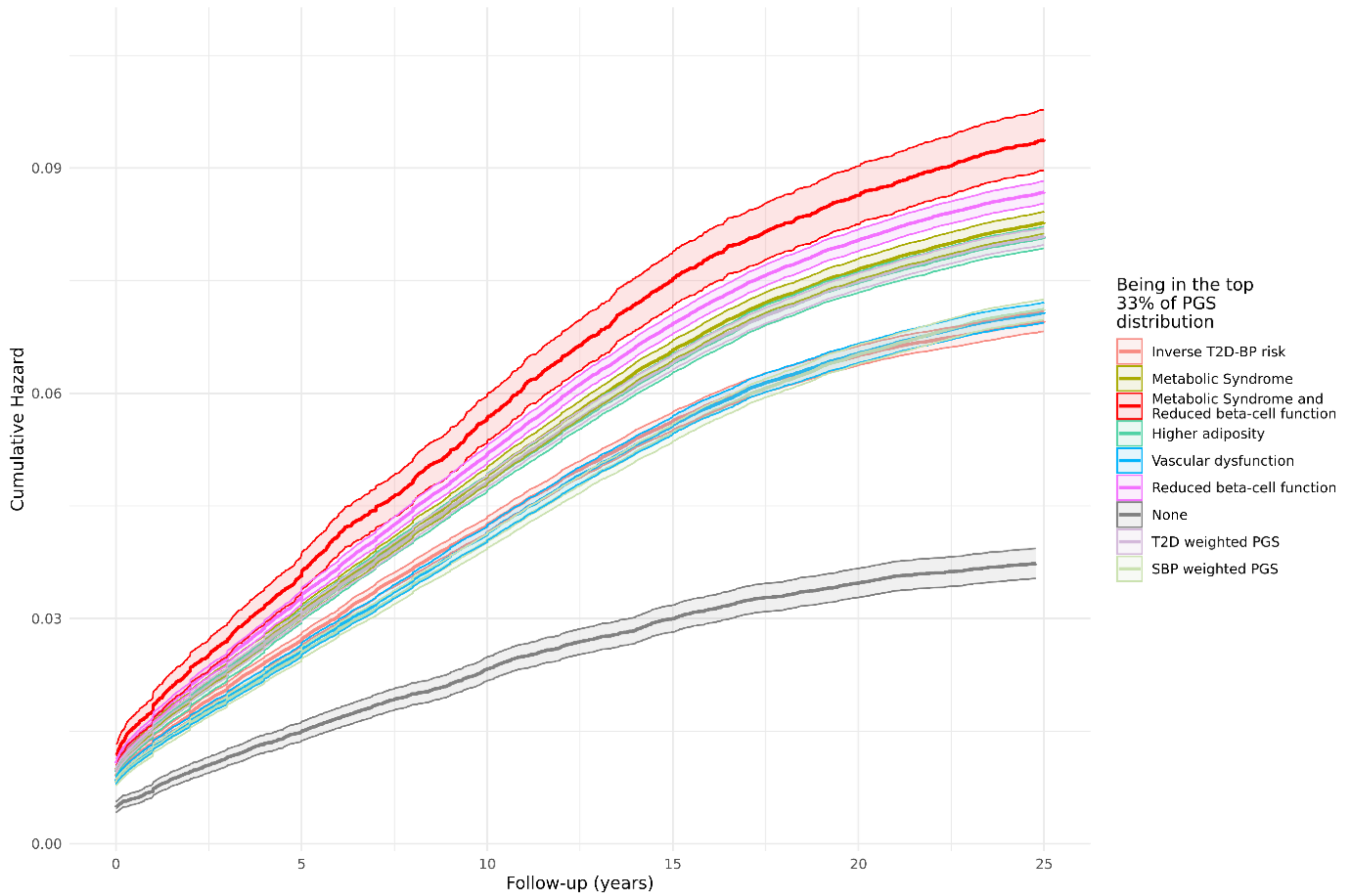

Supplementary Figure 11: Cumulative hazard plot of T2D-BP comorbidity stratified by being in the top 33 percent of unweighted partitioned PGS. PGS are unweighted based on the clustering, alongside traditional P+T weighted PGS for T2D (magenta) and SBP (sage) with 95% CI. Shaded areas represent the 95% CI of the two-sided estimated cumulative hazard from the Cox proportional hazards model.

Legend:

T2D = Type 2 Diabetes; BP = Blood Pressure; SBP = Systolic Blood Pressure; PGS = Polygenic Score; P+T = pruning-and-thresholding; CI = Confidence Interval

2. Supplementary Tables

Supplementary Table 1: Baseline characteristics of the UK BioBank dataset. 17
Supplementary Table 2: Reciprocal risk prediction of Polygenic Scores (PGS) on the risk of high BP and T2D using *comorbidPGS*. 18
Supplementary Table 3: The 15 genes which colocalise in Thyroid tissue and are enriched in cCRE in follicular cells by clusters. 19
Supplementary Table 4: Genes which colocalise in Adipose Subcutaneous, Skin (sun exposed) lower leg, Nerve Tibial, and Artery Tibial tissues and are enriched in cCRE in top cell types per tissue by clusters. 20
Supplementary Table 5: Summary table depicting the risk of T2D-BP comorbidity based on being in the top 10% (top) and top 33% (bottom) of cluster PGSs and weighted PGS as controls in the UKB. 21

Supplementary Table 1: Baseline characteristics of the UK BioBank dataset.

Characteristics	Females	Males	All
Genotypic info	249,297	209,950	459,247
Mean Age	57.09 (7.954) *	57.5 (8.122) *	57.27 (8.034)
Individuals on blood pressure lowering medication	43,042 (17.27%) ⁺	52,670 (25.09%) ⁺	95,712 (20.84%)
Mean BMI	27.01 (5.142) *	27.85 (4.242) *	27.4 (4.77)
Mean SBP	140.1 (22.02) *	146.9 (20.18) *	143.2 (21.46)
Mean DBP	82.29 (11.55) *	86.46 (11.42) *	84.2 (11.68)
Mean PP	57.84 (15.94) *	60.43 (14.37) *	59.03 (15.29)
Hypertensive patients	102,755 (41.22%) ⁺	114,844 (54.70%) ⁺	217,599 (47.38%)
T2D patients	13,136 (5.27%) ⁺	20,310 (9.67%) ⁺	33,446 (7.28%)
Comorbid patients (having Hypertension and T2D)	9,326 (3.74%) ⁺	15,865 (7.56%) ⁺	25,191 (5.49%)

Legend:
BMI = body-mass index; SBP = systolic blood pressure; DBP = diastolic blood pressure; PP = pulse pressure; T2D = Type 2 Diabetes
* = statistical significance in mean difference between sexes using two-sided unpaired Student's t-test;
⁺ = statistically significant difference in contingency between sexes using two-sided Chi-square tests

Supplementary Table 2: Reciprocal risk prediction of Polygenic Scores (PGS) on the risk of high BP and T2D using *comorbidPGS*. Two-sided linear regression for quantitative phenotypes, two-sided logistic regression for binary phenotypes.

PGS on the original Phenotype (P_o-PGS)	Target phenotype P_t	Statistical methods	N_{cases}	$N_{controls}$	Effect (Beta for linear regression or OR for logistic)	Standard Error	Lower CI	Upper CI	P-value
T2D	SBP	Linear regression	NA	458822	0.90	0.029	0.84	0.95	**9.40E-204**
T2D	DBP	Linear regression	NA	458834	0.37	0.017	0.34	0.41	**1.83E-106**
T2D	PP	Linear regression	NA	458822	0.52	0.020	0.48	0.56	**3.96E-143**
SBP	T2D	Logistic regression	33447	425802	1.07		1.06	1.09	**9.36E-35**
DBP	T2D	Logistic regression	33447	425802	1.01		0.999	1.02	0.062
PP	T2D	Logistic regression	33447	425802	1.07		1.06	1.08	**8.02E-31**
SBP	SBP	Linear regression	NA	458822	3.08	0.029	3.03	3.14	**0**
DBP	DBP	Linear regression	NA	458834	1.81	0.017	1.78	1.85	**0**
PP	PP	Linear regression	NA	458822	2.22	0.020	2.18	2.26	**0**
T2D	T2D	Logistic regression	33447	425802	1.78		1.76	1.80	**0**

Legend:

Bold P-value are below the threshold for Bonferroni multiple testing correction ($P < 0.005$)

Beta represents the change of BP in mmHg per one-unit increase in PGS. OR represents the change in T2D odds per one-unit increase in PGS.

PGS = Polygenic Score; BP = Blood Pressure; T2D = Type 2 Diabetes; DBP = Diastolic blood pressure; PP = pulse pressure; SBP = systolic blood pressure; OR = odds ratio; CI = confidence interval (95%); NA = not applicable

Supplementary Table 3: The 15 genes which colocalise in Thyroid tissue and are enriched in cCRE in follicular cells by clusters.

High adiposity cluster	Reduced beta-cell function cluster
CD8A	*CAMK1D*
SAE1	*ACE*
SAE1	*COMMD2*
	DCAF7
	DDX42
	GSAP
	KCNH6
	MAP3K3
	RNF13
	STRADA
	TM4SF4
	WWTR1
	WWTR1-AS1

Legend:
Using Bayesian colocalization (two-sided; PP.H4 > 0.8 and PP.H3 < 0.5).
Bold genes are present in multiple clusters, and differentially up-regulated genes in Thyroid tissue
Underlined genes are found in the *metascape* pathway analysis:
GO:0016567 protein ubiquitination   *GO:0031401 positive regulation of protein*

Supplementary Table 4: Genes which colocalise in Adipose Subcutaneous, Skin (sun exposed) lower leg, Nerve Tibial, and Artery Tibial tissues and are enriched in cCRE in top cell types per tissue by clusters.

Inverse T2D-BP risk cluster	Metabolic Syndrome cluster	High adiposity cluster	Vascular dysfunction cluster	Reduced beta-cell function cluster
**Genes which colocalise in Adipose Subcutaneous tissue & are enriched in cCRE in Adipocyte cell type**				
	MSANTD1			*MAP3K3*
	MRPL18			*STRADA*
**Genes which colocalise in Artery Tibial tissue & are enriched in cCRE in Smooth Muscle cell type**				
TBX18-AS1	*COPZ2*	*FUT11*	*SLC20A2*	*DCAF7*
	PRR15L	*ENSG000000279088 (TEC)*		*DDX42*
	SMUG1			*MAP3K3*
	ENSG00000257534 (lncRNA)			*PCBD1*
**Genes which colocalise in Nerve Tibial tissue & are enriched in cCRE in Schwann cell type**				
				LIMD2
				RNF13
				STRADA
				ENSG00000279369 (TEC)
**Genes which colocalise in Skin (Sun+) lower leg tissue & are enriched in cCRE in Keratinocyte cell type**				
				DDX42
				MAP3K3

Legend:
Using Bayesian colocalization (two-sided; PP.H4 > 0.8 and PP.H3 < 0.5).
cCRE = candidate cis-regulatory elements
Underlined genes are found in the *metascape* pathway analysis:
M30115 Transcription Factor Targets: PCGF1 Target Genes

Supplementary Table 5: Summary table depicting the risk of T2D-BP comorbidity based on being in the top 10% (top) and top 33% (bottom) of cluster PGSs and weighted PGS as controls in the UKB.

**Top 10% of the PGS for**	N individuals	T2D cases (%)	Hypertension cases (%)	T2D-BP comorbidity (%)	RR T2D	RR Hypertension	RR T2D-BP comorbidity
Weighted Pulse Pressure	45,925	7.90%	52.40%	6.30%	1.08	1.11	1.14
Weighted Diastolic Blood Pressure	458,489	7.30%	47.40%	5.50%	1.00	1.00	1.00
Weighted Systolic Blood Pressure	45,924	8.10%	58.40%	6.80%	1.11	1.23	1.24
Weighted T2D	45,925	15.90%	51.30%	11.80%	2.19	1.08	2.16
**Reduced beta-cell function**	45,906	11.30%	49.90%	8.51%	1.55	1.05	1.55
**Vascular dysfunction**	45,915	7.90%	52.90%	6.26%	1.08	1.12	1.14
**Higher adiposity**	45,913	9.80%	50.60%	7.48%	1.34	1.07	1.36
**Metabolic Syndrome**	45,891	10.10%	52.10%	7.88%	1.38	1.10	1.44
**Inverse T2D-BP risk**	45,895	8.10%	43.00%	5.73%	1.11	0.91	1.05
Metabolic Syndrome & Higher adiposity	4,605	13.10%	55.00%	10.20%	1.80	1.16	1.87
Metabolic Syndrome & Vascular dysfunction	4,670	11.00%	57.50%	8.90%	1.52	1.21	1.63
**Metabolic Syndrome & Reduced beta-cell function**	4,641	15.10%	54.60%	11.66%	2.07	1.15	2.13
Metabolic Syndrome & Higher adiposity & Reduced beta-cell function	470	18.90%	56.40%	14.30%	2.60	1.19	2.60
Whole cohort	459,247	7.28%	47.38%	5.49%	1.00	1.00	1.00

**Top 33% of the PGS for**	N individuals	T2D cases (%)	Hypertension cases (%)	T2D-BP comorbidity (%)	RR T2D	RR Hypertension	RR T2D-BP comorbidity
Weighted Pulse Pressure	153,082	7.80%	50.50%	6.00%	1.07	1.07	1.10
Weighted Diastolic Blood Pressure	459,069	7.30%	47.40%	5.50%	1.00	1.00	1.00

Weighted Systolic Blood Pressure	153,082	7.80%	54.20%	6.30%	1.07	1.14	1.14
Weighted T2D	153,082	7.80%	54.20%	6.30%	1.07	1.14	**1.60**
**Reduced beta-cell function**	153,042	9.60%	48.80%	7.20%	1.32	1.03	1.31
**Vascular dysfunction**	153,068	7.70%	50.70%	6.00%	1.05	1.07	1.09
**Higher adiposity**	152,989	8.70%	49.40%	6.70%	1.20	1.04	1.22
**Metabolic Syndrome**	153,080	9.00%	50.50%	6.90%	1.23	1.07	1.26
**Inverse T2D-BP risk**	153,037	7.80%	44.50%	5.60%	1.07	0.94	1.03
Metabolic Syndrome & Higher adiposity	51,481	10.50%	52.30%	8.20%	1.45	1.10	1.49
Metabolic Syndrome & Vascular dysfunction	51,446	9.30%	53.60%	7.40%	1.28	1.13	1.35
**Metabolic Syndrome & Reduced beta-cell function**	51,446	9.30%	53.60%	7.40%	1.28	1.13	**1.62**
Metabolic Syndrome & Higher adiposity & Reduced beta-cell function	17,605	13.50%	53.80%	10.50%	1.86	1.14	1.92
Whole cohort	459,247	7.28%	47.38%	5.49%	1.00	1.00	1.00

Legend: T2D = Type 2 Diabetes; SBP = Systolic blood pressure; PGS = Polygenic Score; T2D-BP represents the individuals with both T2D and hypertension as reported conditions
Relative risk (RR) is calculated as the ratio of the proportion of cases within a cluster subgroup to the proportion of cases in the overall population, $RR=(Prev_{\text{cases in cluster}})/(Prev_{\text{cases overall}})$.
