# Supplementary material for: Partitioned polygenic scores show mechanistic heterogeneity in type 2 diabetes and hypertension comorbidity
Source: Nat Commun. 2026 Feb 9;17:1446. doi: 10.1038/s41467-025-67449-2 (PMC12886974; doi:10.1038/s41467-025-67449-2)
Supplement: Supplementary file 3 — Reporting Summary [file 41467_2025_67449_MOESM3_ESM.pdf]

nature portfolio

Corresponding author(s): Prof Inga Prokopenko, NCOMMS-25-16791B

Last updated by author(s): Nov 17, 2025

Reporting Summary

Nature Portfolio wishes to improve the reproducibility of the work that we publish. This form provides structure for consistency and transparency in reporting. For further information on Nature Portfolio policies, see our Editorial Policies and the Editorial Policy Checklist.

Statistics

For all statistical analyses, confirm that the following items are present in the figure legend, table legend, main text, or Methods section.

n/a	Confirmed	
☐	☒	The exact sample size (n) for each experimental group/condition, given as a discrete number and unit of measurement
☒	☐	A statement on whether measurements were taken from distinct samples or whether the same sample was measured repeatedly
☐	☒	The statistical test(s) used AND whether they are one- or two-sided<br>*Only common tests should be described solely by name; describe more complex techniques in the Methods section.*
☐	☒	A description of all covariates tested
☐	☒	A description of any assumptions or corrections, such as tests of normality and adjustment for multiple comparisons
☐	☒	A full description of the statistical parameters including central tendency (e.g. means) or other basic estimates (e.g. regression coefficient) AND variation (e.g. standard deviation) or associated estimates of uncertainty (e.g. confidence intervals)
☐	☒	For null hypothesis testing, the test statistic (e.g. F, t, r) with confidence intervals, effect sizes, degrees of freedom and P value noted<br>*Give P values as exact values whenever suitable.*
☒	☐	For Bayesian analysis, information on the choice of priors and Markov chain Monte Carlo settings
☐	☒	For hierarchical and complex designs, identification of the appropriate level for tests and full reporting of outcomes
☐	☒	Estimates of effect sizes (e.g. Cohen's d, Pearson's r), indicating how they were calculated

Our web collection on statistics for biologists contains articles on many of the points above.

Software and code

Policy information about availability of computer code

Data collection	No software used for data collections.
Data analysis	We used R version 4.2 for data analysis, alongside R packages including ldsc v1.0.1 for genetic heritability/correlation, plink v1.9 and comorbidPGS v1 to build polygenic score, imputeSCOPA v0.1 for imputation in the hiearachical clustering, bNMF v1 and pheatmap v1.0.12 for clustering, coloc v5.2 for colocalisation. MRClust (github access, unreleased) for Mendelian Randomization sensitivity analysis.<br>The main code used in this analysis can be found in this GitHub repository: https://doi.org/10.5281/zenodo.17448298.

For manuscripts utilizing custom algorithms or software that are central to the research but not yet described in published literature, software must be made available to editors and reviewers. We strongly encourage code deposition in a community repository (e.g. GitHub). See the Nature Portfolio guidelines for submitting code & software for further information.

Data

Policy information about availability of data

All manuscripts must include a data availability statement. This statement should provide the following information, where applicable:

- Accession codes, unique identifiers, or web links for publicly available datasets
- A description of any restrictions on data availability
- For clinical datasets or third party data, please ensure that the statement adheres to our policy

The GWAS used in this study are all publicly available and listed in Supplementary Data 2. The UK Biobank Resource (UKB, https://ukbiobank.ac.uk/) was accessed

using the Application Number 236. GTEx (https://www.gtexportal.org/home/downloads/adult-gtex/qtl) and TIGER (https://tiger.bsc.es/assets/summary_statistics_tiger.tar.gz) eQTLs data used in this study are publicly available via these links, respectively. Data from the ABOS cohort are not publicly available, since they are subject to national data protection laws and restrictions imposed by the ethics committee to ensure data privacy of the study participants. Data can be accessed through an individual project agreement with the principal investigator of the University Hospital of Lille (Lille, France), Prof François Pattou. The ATAC-seq data from CATLAS are publicly available and can be accessed via the following link: https://catlas.org/humanenhancer/data/.

Research involving human participants, their data, or biological material

Policy information about studies with human participants or human data. See also policy information about sex, gender (identity/presentation), and sexual orientation and race, ethnicity and racism.

Reporting on sex and gender	We made use of multi-ancestry datasets to the fullest extent possible. When robust data across multiple ancestry groups were available, all were included in the analysis. In cases where no suitable alternative was available, analyses were restricted to individuals of genetically inferred European ancestry. All references to ancestry are based on genetically inferred population structure and not on self-reported race or ethnicity. Similarly, the reported sex in this work was determined using genetic data.
Reporting on race, ethnicity, or other socially relevant groupings	We made use of multi-ancestry datasets to the fullest extent possible. When robust data across multiple ancestry groups were available, all were included in the analysis. In cases where no suitable alternative was available, analyses were restricted to individuals of genetically inferred European ancestry. All references to ancestry are based on genetically inferred population structure and not on self-reported race or ethnicity. Similarly, the reported sex in this work was determined using genetic data.
Population characteristics	This study is based exclusively on previously published and publicly available datasets; no new data involving human participants or animals were collected. Hence, no additional ethical approval was required for this research. The UK Biobank dataset in this study derived from genome-wide imputed data, including 459,247 individuals of European ancestry.<br><br>We systematically used covariates to perform association tests, including genetic-inferred sex, genetic-inferred ancestry, age, chip (used by the UK Biobank to genotype individuals), and principal components.
Recruitment	This study is based exclusively on previously published and publicly available datasets; no new data involving human participants or animals were collected. Hence, no additional ethical approval was required for this research.
Ethics oversight	This study is based exclusively on previously published and publicly available datasets; no new data involving human participants or animals were collected. Hence, no additional ethical approval was required for this research.<br>We made use of multi-ancestry datasets to the fullest extent possible. When robust data across multiple ancestry groups were available, all were included in the analysis. In cases where no suitable alternative was available, analyses were restricted to individuals of genetically inferred European ancestry. All references to ancestry are based on genetically inferred population structure and not on self-reported race or ethnicity. Similarly, the reported sex in this work was determined using genetic data. UK Biobank Resource (UKB, https://ukbiobank.ac.uk/) was accessed using the Application Number 236

Note that full information on the approval of the study protocol must also be provided in the manuscript.

Field-specific reporting

Please select the one below that is the best fit for your research. If you are not sure, read the appropriate sections before making your selection.

☒ Life sciences ☐ Behavioural & social sciences ☐ Ecological, evolutionary & environmental sciences

For a reference copy of the document with all sections, see nature.com/documents/nr-reporting-summary-flat.pdf

Life sciences study design

All studies must disclose on these points even when the disclosure is negative.

Sample size	We used 45 publicly available GWAS summary statistics with different sample size as discovery datasets (N>10000). We the used the GTEx, TIGER and ABOS (N=1000; 500; 372) eQTL datasets alongside the CATLAS data (N=30 adults and 15 fetal cells) to characterised the discovered genetic variants. We validated these by using the UK Biobank cohort (N=459,247).
Data exclusions	To mitigate discrepancies in LD patterns between all the datasets used in this study, we limited our analysis to dataset with a majority of individuals from European descents (genetically-inferred).
Replication	We the used the GTEx, TIGER and ABOS (N=1000; 500; 372) eQTL datasets alongside the CATLAS data (N=30 adults and 15 fetal cells) to characterised the discovered genetic variants. We validated these by using the UK Biobank cohort (N=459,247).
Randomization	Randomization per se is not applicable to this study design. However, the large-scale datasets selected in this study prevented us to catch big bias in population structure/prevalence of metabolic conditions.
Blinding	Blinding per se is not applicable to this study design. The only resource with individual-level used is the UK Biobank, which anonymised their data. No real blinding is required for this analysis as the remaining datasets are summary-level (giving information on a gene-level, genetic variant-level without direct link to an individual).

Reporting for specific materials, systems and methods

We require information from authors about some types of materials, experimental systems and methods used in many studies. Here, indicate whether each material, system or method listed is relevant to your study. If you are not sure if a list item applies to your research, read the appropriate section before selecting a response.

Materials & experimental systems

n/a	Involved in the study
☒	☐ Antibodies
☒	☐ Eukaryotic cell lines
☒	☐ Palaeontology and archaeology
☒	☐ Animals and other organisms
☒	☐ Clinical data
☒	☐ Dual use research of concern
☒	☐ Plants

Methods

n/a	Involved in the study
☒	☐ ChIP-seq
☒	☐ Flow cytometry
☒	☐ MRI-based neuroimaging

Plants

Seed stocks	*Report on the source of all seed stocks or other plant material used. If applicable, state the seed stock centre and catalogue number. If plant specimens were collected from the field, describe the collection location, date and sampling procedures.*
Novel plant genotypes	*Describe the methods by which all novel plant genotypes were produced. This includes those generated by transgenic approaches, gene editing, chemical/radiation-based mutagenesis and hybridization. For transgenic lines, describe the transformation method, the number of independent lines analyzed and the generation upon which experiments were performed. For gene-edited lines, describe the editor used, the endogenous sequence targeted for editing, the targeting guide RNA sequence (if applicable) and how the editor was applied.*
Authentication	*Describe any authentication procedures for each seed stock used or novel genotype generated. Describe any experiments used to assess the effect of a mutation and, where applicable, how potential secondary effects (e.g. second site T-DNA insertions, mosiacism, off-target gene editing) were examined.*
